# Generation and analysis of a 29,745 unique Expressed Sequence Tags from the Pacific oyster (*Crassostrea gigas*) assembled into a publicly accessible database: the GigasDatabase

**DOI:** 10.1186/1471-2164-10-341

**Published:** 2009-07-29

**Authors:** Elodie Fleury, Arnaud Huvet, Christophe Lelong, Julien de Lorgeril, Viviane Boulo, Yannick Gueguen, Evelyne Bachère, Arnaud Tanguy, Dario Moraga, Caroline Fabioux, Penelope Lindeque, Jenny Shaw, Richard Reinhardt, Patrick Prunet, Grace Davey, Sylvie Lapègue, Christopher Sauvage, Charlotte Corporeau, Jeanne Moal, Frederick Gavory, Patrick Wincker, François Moreews, Christophe Klopp, Michel Mathieu, Pierre Boudry, Pascal Favrel

**Affiliations:** 1 UMR M100 Ifremer – Université de Caen Basse-Normandie « Physiologie et Ecophysiologie des Mollusques Marins », Centre de Brest, B.P. 70, 29280 Plouzané/IBFA, IFR ICORE 146, Esplanade de la Paix, 14032 Caen Cedex, France; 2 IFREMER CNRS Université de Montpellier 2, UMR 5119 ECOLAG CC 80, Place Eugène Bataillon, 34095 Montpellier cedex 5, France; 3 CNRS, UMR 7144, Adaptation et Diversité en Milieu Marin, Station Biologique de Roscoff, 29682 Roscoff, France; 4 Laboratoire des Sciences de l'Environnement Marin (LEMAR), UMR-CNRS 6539, Institut Universitaire Européen de la Mer, Université de Bretagne Occidentale, Place Nicolas Copernic, 29280, Plouzané, France; 5 Plymouth Marine Laboratory, Prospect Place, West Hoe, Plymouth, Devon PL1 3DH, UK; 6 MPI Molecular Genetics, Ihnestrasse 63-73, D-14195 Berlin-Dahlem, Germany; 7 Institut National de la Recherche Agronomique, INRA-SCRIBE, IFR 140, Campus de Beaulieu, 35000 Rennes, France; 8 National Diagnostics Centre, National University of Ireland Galway, Galway, Ireland; 9 Laboratoire de Génétique et Pathologie, Ifremer La Tremblade, 17390 La Tremblade, France; 10 CEA, DSV, Genoscope, Centre National de Séquençage, 2 rue Gaston Crémieux CP5706 91057 Evry cedex, France; 11 INRA, Sigenae UR875 Biométrie et Intelligence Artificielle, BP 52627, 31326 Castanet-Tolosan Cedex, France

## Abstract

**Background:**

Although bivalves are among the most-studied marine organisms because of their ecological role and economic importance, very little information is available on the genome sequences of oyster species. This report documents three large-scale cDNA sequencing projects for the Pacific oyster *Crassostrea gigas *initiated to provide a large number of expressed sequence tags that were subsequently compiled in a publicly accessible database. This resource allowed for the identification of a large number of transcripts and provides valuable information for ongoing investigations of tissue-specific and stimulus-dependant gene expression patterns. These data are crucial for constructing comprehensive DNA microarrays, identifying single nucleotide polymorphisms and microsatellites in coding regions, and for identifying genes when the entire genome sequence of *C. gigas *becomes available.

**Description:**

In the present paper, we report the production of 40,845 high-quality ESTs that identify 29,745 unique transcribed sequences consisting of 7,940 contigs and 21,805 singletons. All of these new sequences, together with existing public sequence data, have been compiled into a publicly-available Website http://public-contigbrowser.sigenae.org:9090/Crassostrea_gigas/index.html. Approximately 43% of the unique ESTs had significant matches against the SwissProt database and 27% were annotated using Gene Ontology terms. In addition, we identified a total of 208 *in silico *microsatellites from the ESTs, with 173 having sufficient flanking sequence for primer design. We also identified a total of 7,530 putative *in silico*, single-nucleotide polymorphisms using existing and newly-generated EST resources for the Pacific oyster.

**Conclusion:**

A publicly-available database has been populated with 29,745 unique sequences for the Pacific oyster *Crassostrea gigas*. The database provides many tools to search cleaned and assembled ESTs. The user may input and submit several filters, such as protein or nucleotide hits, to select and download relevant elements. This database constitutes one of the most developed genomic resources accessible among Lophotrochozoans, an orphan clade of bilateral animals. These data will accelerate the development of both genomics and genetics in a commercially-important species with the highest annual, commercial production of any aquatic organism.

## Background

Genome research on the Pacific oyster, *Crassostrea gigas*, has been facilitated by the recent development of species-specific tools such as linkage maps [1,2], large-insert libraries [3], a public clearing-house [4], and gene expression profiles [5-7]. Several factors motivate further development of genomic resources for *C. gigas*: (I) Because this species has the highest annual production of any aquatic organism, *C. gigas *has been the subject of a great deal of research to elucidate the molecular basis underlying the physiological and genetic mechanisms of economically-relevant traits. (II) The Pacific oyster's phylogenic position in the Lophotrochozoa, an understudied clade of bilaterian animals, makes molecular data on *C. gigas *highly relevant for studies of genome evolution. (III) Oysters play an important role as sentinels in estuarine and coastal marine habitats where increasing human activities exacerbate the impacts of disease and stress in exploited populations. (IV) *C. gigas *can be an invasive species when introduced into new habitats [8]. As a result, the Pacific oyster is becoming an attractive model species for genome-related research activities focusing on comparative immunology [*e.g*. [9-11]], disease ecology [*e.g*. [12-14]], stress response to pollutants and parasites [*e.g*. [15]], developmental and reproductive physiology [*e.g*. [16,17]] and evolutionary genetics [*e.g*. [18-20]].

The genomic strategies currently employed for the identification of novel and previously-characterized genes affecting phenotypes of interest in the Pacific oyster include the identification of quantitative trait loci (QTL), and high-throughput studies of gene expression [21]. QTL mapping of genetic variation affecting, for example, resistance to summer mortality [22] or hybrid vigor [6] requires a large number of mapped molecular markers and testing for associations between marker genotypes and phenotypes to identify chromosomal regions harbouring genes that directly affect the trait of interest. Recently developed BAC libraries and fingerprinting [3] (P. Gaffney, Pers. Com.), have facilitated fine mapping of such regions, and ultimately specification of marker position on the genetic linkage map, allowing gene-assisted selection. Functional genomic approaches are also required for gene-expression profiling experiments such as macroarrays [17], microarrays [7], SAGE (Serial Analysis of Gene Expression), MPSS (Massively Parallel Signature Sequencing) [6], or technologies addressing single genes, such RT-qPCR (real-time quantitative PCR). These techniques have potential applications in ecological monitoring [23], evaluating oyster broodstock for selective breeding and understanding of gene regulation involved, for example, in the molecular pathways associated with responses to stress or pathogens.

In the present paper, we report the generation and analysis of 47,889 ESTs by sequencing clones from the Network of Excellence "Marine Genomics Europe" (MGE) normalized gonad cDNA library (partially published in [24]), and two other projects: I) the Genoscope project (CEA Evry, France) and II) the European Aquafirst project (Table 1). The objective of the Genoscope project (EST sequencing from *Crassostrea gigas*) was to substantially expand genomic information on oysters by sequencing ESTs from: (I) a normalized "hemocyte" cDNA library constructed with mRNA from bacteria-challenged and unchallenged hemocytes, and (II) ESTs from an "all developmental stages and Central Nervous System (CNS)" normalized cDNA library derived from mRNA extracted from all embryonic and larval stages, as well as from adult visceral ganglia. The European "Aquafirst" project that uses genetic and functional genomic approaches to develop summer mortality resistance markers in oysters, produced ESTs by suppression subtractive hybridization between Resistant and Sensitive oyster lines in six different tissues [25]. To maximize the utility of these collections, ESTs from all of these efforts, together with those in public databases (*e.g*. [26]), have been assembled in a unique public database: the GigasDatabase http://public-contigbrowser.sigenae.org:9090/Crassostrea_gigas/index.html containing 29,745 unique sequences.

**Table 1:** Summary statistics of the Pacific oyster cDNA libraries.

Description	Tissue	Vector used	No. of sequences retrieved	No. of valid sequences	Average length insert (bp)
cDNA gonad *	gonad	PAL32CV	12162	8809	511
cDNA embryos and larvae and central nervous system (GENOSCOPE)	embryos, larvae, CNS	pAL17.3	13191	12730	618
cDNA hemocytes (GENOSCOPE)	hemocyte	pBluscriptIISK+	14472	13773	415
cDNA digestive gland subtracted library (AQUAFIRST)	digestive gland	PCR2.1	1536	1362	428
cDNA mantle-edge subtracted library (AQUAFIRST)	mantle-edge	PCR2.1	1536	1343	405
cDNA hemocyte subtracted library (AQUAFIRST)	hemocyte	PCR2.1	1152	125	291
cDNA gonad subtracted library (AQUAFIRST)	gonad	PCR2.1	768	559	382
cDNA muscle subtracted library (AQUAFIRST)	muscle	PCR2.1	1536	1117	312
cDNA gills subtracted library (AQUAFIRST)	gills	PCR2.1	1536	1027	359
Total			47889	40845	Mean: 413

* Library partially published in Tanguy et al.[25] with 1894 sequenced clones.

This resource is highly valuable for identifying important gene networks controlling physiological processes, it facilitates the development of molecular markers for the construction of a reference genetic map, and it allows large-scale, expression-profiling experiments using microarrays. These tools will be useful to advance our knowledge of the genetic and physiological bases of development, reproduction, immunology, and associated processes that are important for oyster aquaculture. Finally, this work will be very useful for the annotation phase of the entire oyster genome, the principal objective of an international community of oyster biologists [27] that will provide a critical point of comparison for understanding the early diversification of animals and their genome, as has been recently proposed for the gastropod snail *Lottia gigantea *http://genome.jgi-psf.org/Lotgi1/Lotgi1.home.html.

## Construction and content

### 1. Biological samples

#### 1.1. Resistant and Sensitive oysters for the subtractive libraries

Resistant (R) and Susceptible (S) oyster families were produced, through divergent selection for high or low survival of summer mortality, as fully described previously [25]. After completing rearing of oysters through larval development in the IFREMER hatchery in La Tremblade (France, July 2004), we transferred juvenile oysters to the nursery of Bouin (Vendée, France) until March 2005, when the oysters were deployed in the field at Fort Espagnol (South Brittany, France). We collected samples of gonad, muscle, digestive gland, hemocytes, mantle-edge, and gills from 12 R and 12 S oysters on two dates (May 25 and June 6, 2005) and individual tissues from each line were pooled at each sampling date.

#### 1.2. Biological material for the "all developmental stages and central nervous system" cDNA library

Mature, wild oysters, collected on the Atlantic coast of Brittany (France), were spawned and reared in captivity as described in [28]. From this pool, we sampled various developmental stages, which we identified microscopically: oocytes before fertilization, 4-cell and 8-cell embryos (1 and 2 hours post-fertilization [hpf], respectively), morula (3 hpf), blastula (5 hpf), gastrula (7 hpf), trochophore larvae (16 hpf) and D-larvae (2 days post-fertilization [dpf]), early veliger larvae (7 dpf), later veliger larvae (14 dpf), pediveliger larvae (18 dpf), and spat after metamorphosis (27 dpf). We extracted total mRNA from one million oysters from each developmental stage from oocyte to trochophore, and 250,000 from later stages, and from the visceral ganglia microscopically dissected from 10 wild, adult oysters

#### 1.3. Biological material for "hemocytes" cDNA library

We sampled hemocytes from six adult oysters exposed to each of 24 experimental conditions (total n = 144), all combinations of four kinds of bacterial challenge, two times post-challenge, and oysters collected from three geographic origins: Atlantic coast (La Tremblade), Normandie (Bay des Veys) and Mediterranean Sea (Thau lagoon). We performed the bacterial challenges by immersing oysters in seawater containing (I) live, non-virulent *Micrococcus luteus *and *Vibrio tasmaniensis *(2.5 × 10^8 ^bacteria/L for each strain), (II) live, virulent *Vibrio splendidus *(5 × 10^8 ^bacteria/L), (III) a mix of heat-killed, virulent *Vibrio splendidu*s and *Vibrio aesturianaus *(2.5 × 10^8 ^bacteria/L for each strain) and (IV) unchallenged oysters. For each condition, we collected hemolymph at 22 and 24 h post challenge from the pericardial cavity through the adductor muscle. We isolated hemocytes separately from these samples (24 experimental conditions) by centrifugation at 700 g for 10 min (4 C) and discarded the plasma. Hemocytes were further subjected to several experimental procedures (see below).

### 2. RNA preparation

Total RNA was isolated using Trizol reagent (Gibco BRL) at a concentration of 1 ml/30 mg of tissue. For SSH experiments and hemocyte samples, we isolated polyadenylated RNA using the Quickprep micro mRNA purification kit (Amersham). We measured RNA concentration with a spectrophotometer at 260 nm using the conversion factor 1 OD = 40 μg/ml RNA, and RNA quality was determined using a Bioanalyser 2100 (Agilent).

### 3. cDNA library construction and sequencing of the clones

#### 3.1. Construction of subtractive libraries

The mRNA from each pool of tissue RNA (6 tissues in total) was used as the template for SSH following the PCR-select cDNA subtraction kit procedure (Clontech). Hybridization and subtraction steps were carried out in both directions, *i.e*. for forward subtraction the Resistant (R) sample (tester) was subtracted from the Susceptible (S) sample (driver) and *vice versa *for reverse subtraction. The PCR products from both subtractions were cloned into pCR 2.1^® ^TOPO plasmid using TOP10 One Shot ^® ^competent cells for transformation (Invitrogen).

#### 3.2. Construction of "all Developmental stages and central nervous system" cDNA library

Total RNA (0.3 μg) from a pool of various developmental stages and visceral ganglia was used for ds cDNA synthesis and amplification using the SMART approach. Recovered cDNA was equalized using the Duplex-Specific nuclease (DSN)-based normalization method [29]. Efficiency of normalization was measured by real-time PCR. A severe decrease (shift of 11 amplification cycles) in actin relative copy number was measured in the normalized cDNA sample, when compared to the non normalized sample. Resulting, normalized cDNA was then amplified, directionally cloned into pAL17.3 plasmid (Evrogen, Moscow), and was used to transform the XL1-Blue *E. coli *strain (Stratagene) to generate a cDNA library of 5 × 10^5 ^independent clones.

#### 3.3 Construction of oyster "hemocytes" cDNA library

The cDNA library was built by GATC Biotech AG (Germany). Briefly, 1 μg of total RNA, composed of equimolar concentrations from oyster hemocytes from each of the 24 experimental conditions, was used for ds cDNA synthesis. The normalization method consisted of denaturation and controlled, incomplete reassociation of double-stranded cDNA, followed by selective cloning of the cDNA corresponding to the single-stranded, normalized fraction. Efficiency of normalization was measured by non-radioactive, Reverse Northern blot analysis of normalized and non-normalized cDNA. Briefly, an array containing 96 randomly-selected clones derived from the non-normalized cDNA library was hybridized with normalized and non-normalized cDNA. Comparison of homogenization of hybridization signals from the normalized probe to the non-normalized probe indicated the efficiency of normalization (data not shown). Normalized cDNA was then amplified, directionally cloned into pBluescriptIISK(+) plasmid (Stratagene), and was used to transform *E. coli *XL1-BlueMRF' (Stratagene) to generate a normalized cDNA library of 8.3 × 10^4 ^independent clones.

#### 3.4. Sequencing

For subtracted libraries and the normalized cDNA gonad library [24], the clones were sequenced at the Max Planck Institute platform (Berlin, Germany) using an ABI 3730 automatic capillary sequencer, the ABI Big Dye Terminator sequencing kit and universal primer. For the two other normalized cDNA libraries, sequencing was performed at the Genoscope facility (CEA Evry, France) as described above.

### 4. Database, sequence processing and contig assembly

**Figure 1:**
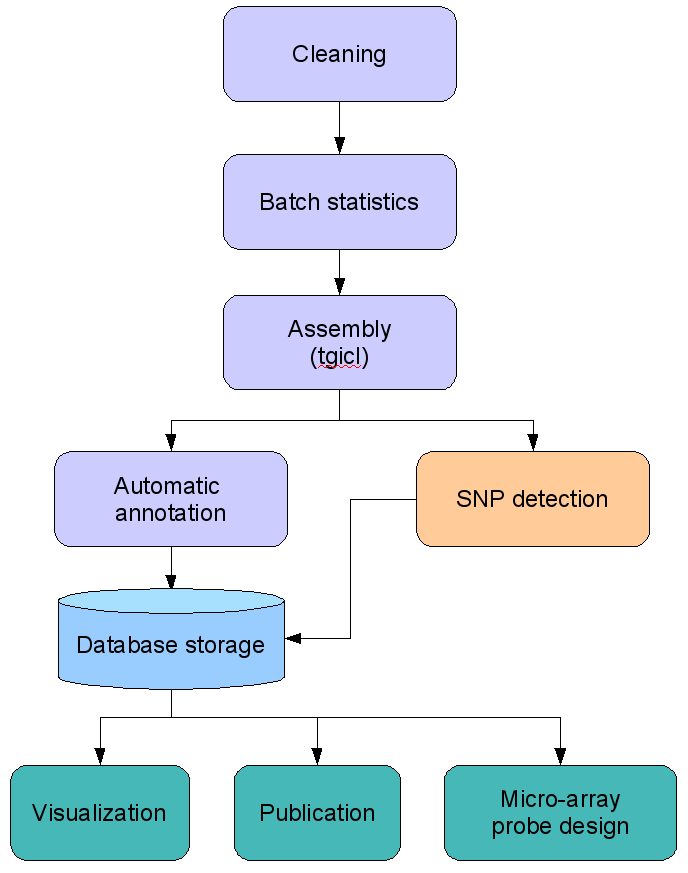
**Processing chain of the GigasDatabase**. The data resources of the GigasDatabase includes cleaning processes, batch statistics, assembling sequences into contigs, annotation of the contigs, visualization of the contigs, and summary statistics concerning each library.

The data files produced were processed by SIGENAE; documentation of procedures is available on the SIGENAE website http://www.sigenae.org/index.php?id=9. The resource data flow has been compiled in the GigasDatabase http://public-contigbrowser.sigenae.org:9090/Crassostrea_gigas/download, as shown in Figure 1.

The sequences were first cleaned up from vector and adaptator sequences. Repeats and contaminants were removed by comparison with several sequence databases such as Univec, Yeast and *E. coli *genomes. The PolyA site was identified by its relative position to the vector multiple-cloning site. In the 30 bases preceding the polyA site, we searched for putative polyadenylation signals (AATAAA and ATTAAA) [30]. Valid sequences, that had a PHRED score over 20 on at least 100 bp, were submitted to the EMBL-EBI Nucleotide Sequence database http://www.ebi.ac.uk/embl/. Because of the large number of sequences, we used a two-step process to assemble these sequences into contigs. The first step built clusters of sequences sharing at least 75 bp with an identity rate of 96% using MegaBlast [31]. The second step constructed coherent contigs from the previous clusters using CAP3 [32], at the recommended stringency of 40 bp overlap with 90% sequence identity. Once the contigs were built and their annotation completed, all data were loaded in a locally-adapted Ensembl database.

To obtain as much information as possible concerning the contigs, we performed similarity searches with BlastX http://blast.ncbi.nlm.nih.gov/Blast.cgi using a variety of databases: UniProt/SwissProt, UniProt/TrEMBL, ProDom (protein domains), UniGene Human Clusters, UniGene taxon specific Clusters, TIGR Taxon Specific Clusters, Ensembl specific transcripts (cDNA), and other Sigenae Contigs. We then loaded the annotations into the GigasDatabase. We identified putative open-reading frames (ORFs) by choosing the longest possible translation into amino acid sequence, using Emboss sixpack http://bioweb2.pasteur.fr/docs/EMBOSS/sixpack.html. Sequences with ORFs smaller than 100 codons (300 bp) were removed from the dataset.

### 5. Gene ontology annotation

To link ESTs with BlastX hits with putative function, we annotated all of them according to gene ontology (GO) terms by using the program KAAS (KEGG Automatic Annotation Server: http://www.genome.jp/tools/kaas/), which provides functional annotation of genes by Blast comparisons (single best hit) against the manually curated KEGG Genes database [33]. The top level consists of the following categories: metabolism, genetic information processing, environmental information processing, and cellular processes. The second level divides these functional categories into finer sub-categories [34]. The distribution of genes in each of the main ontology categories was examined, and the percentages of unique sequence in each of the assigned GO terms were computed.

### 6. in silico mining of microsatellites and SNPs

We searched a set of 56,327 unique sequences for microsatellite markers using SRR finder (http://www.maizemap.org/bioinformatics/SSRFINDER/SSR_Finder_Download.html; [35]) with a minimum repeat of 4. This provided a table of raw data which was then exported to MS Excel^® ^to calculate the number of di-, tri-, and tetra-nucleotide repeats, their respective lengths, and starting positions. Putative SNPs were detected using the Pupasuite v1.0 (http://pupasuite.bioinfo.cipf.es/, [36]). The 56,327 ESTs were assembled in 7,940 contigs wherein putative SNPs were identified and characterized to calculate the percentage of each type of mutation (ts/tv, synonymous/non-synonymous).

## Utility and discussion

### 1. Generation of ESTs

To augment the 1894 recently-published sequences from the normalized cDNA libraries produced by the "Marine Genomic Europe" program [24], we sequenced a novel part of the gonad library. In addition, we also constructed two new, directionally-cloned, normalized oyster cDNA libraries: one including all developmental stages from embryos to larvae and visceral ganglia, and one from bacteria-challenged and unchallenged hemocytes. Single-pass sequencing produced sequences from the 5' regions of mRNA from each library, resulting in 12,162 sequences, 13,191 sequences, and 14,472 sequences, respectively (Table 1).

Finally, to increase the number of genes characterized that are related to summer mortality [13,14,17], we constructed libraries from six different tissues (digestive gland, mantle-edge, hemocytes, gonad, muscle, and gills) using Suppression Subtractive Hybridization (SSH) between selectively-bred Resistant (R) and Sensitive (S) oyster lines [25], by subtracting in both directions (R-S and S-R). These SSH libraries produced of a total of 8,064 sequences, with approximately 1,000 sequences per library (Table 1). All 47,889 sequences were subjected to pre-processing to eliminate poor-quality sequences and remove cloning-vector sequences. After removing clones with very short inserts or no inserts, and those with poor sequence quality, we obtained a total of 40,845 (85.3%) high-quality ESTs with an average length of 413 bp (Table 1). All EST sequences have been deposited in GenBank with the accession numbers [AM857416-AM869575] for gonad library, [CU998430–CU999999; FP000001–FP012228] for hemocyte library, [CU983906–CU998429] for developmental stages and visceral ganglia library, and [CU681473–CU681818; CU682012–CU682338; CU683068–CU683823; CU683828–CU683864; CU684729–CU686587; FP89705–FP89949] for the SSH libraries.

### 2. Contig assembly of the ESTs

The GigasDatabase http://public-contigbrowser.sigenae.org:9090/Crassostrea_gigas/index.html has been used for sequence processing, contig assembly, annotation, and project data hosting. All sequences, including public EST and mRNA sequences, as well as other data and results, can be accessed through the database. After assembly of the 40,845 new, valid ESTs and the 9,548 published sequences from GenBank into contigs, the database consists of 7,940 contigs and 21,805 singletons (Table 2). Thus, the GigasDatabase now contains 29,745 unique sequences from *C. gigas *with an average length of 798 bp (Table 2). As the NCBI actually contains 1325 sequences of *C. gigas *in the "nucleotide section", the release of these newly-generated ESTs will consequently improve the knowledge of sequences for this species.

**Table 2:** Summary statistics of the ESTs generated from the Pacific oyster *Crassostrea gigas *available in the GigasDatabase.

Feature	Value
Number of high quality ESTs (new ESTs + public)	56327 (40845 + 15482)
Average length of high quality ESTs (bp)	798
Number of contigs	7940
Number of ESTs in contigs	34522
Number of singletons	21805
Number of unique sequences	29745

Of the 7,940 contigs, 4,208 contained 2 ESTs (53%), 1,588 contained 3 ESTs (20%), 794 contained 4 ESTs (10%), 397 contained 5 ESTs (5%), and relatively few sequences contained more than 6 ESTs (11%) (Table 3). These results indicate that most of the clusters were small, reflecting a high efficiency in normalization of the cDNA libraries.

**Table 3:** BlastX searches and contig analysis for the complete collection of oyster contigs, based on GigasDatabase EST clustering.

Number of unique sequences	29745
Number of unique sequences with BlastX hits	12790
Percentage of unique sequences with BlastX hits	43%
Number of contigs containing:	
2 ESTs	4208
3 ESTs	1588
4 ESTs	794
5 ESTs	397
> 6 ESTs	873

**Figure 2:**
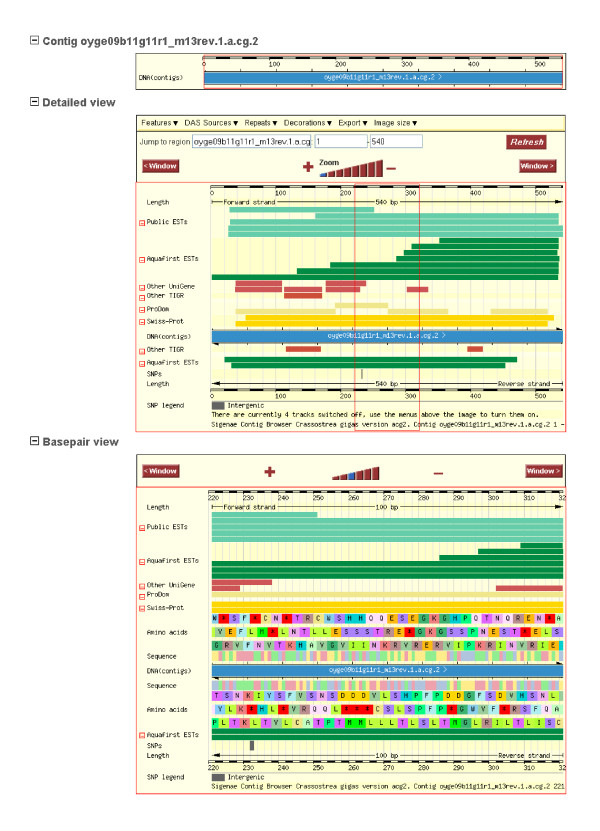
**Graphical view of the contig by "ContigView" available in GigasDatabase**. The ContigView screen gives a graphical overview of the contig structure. Each sequence is represented as a line, and colors indicate the type of sequence. The first level corresponds to the sequence fragment overview. The second level is the detailed view of the individual sequences belonging to the contig. The red frame represents the visualized section on the third level. The third level is the base-pair view of the DNA contigs.

A graphical user interface permits the visualization of the data with different views, such as "ContigView" which gives a graphical overview of the contig structure and similarity annotations. Each sequence or similarity feature is represented as a line. The color of the line gives an indication of the type of sequence and all lines are described on the left of each panel (Figure 2).

### 3. Putative identities of ESTs

**Figure 3:**
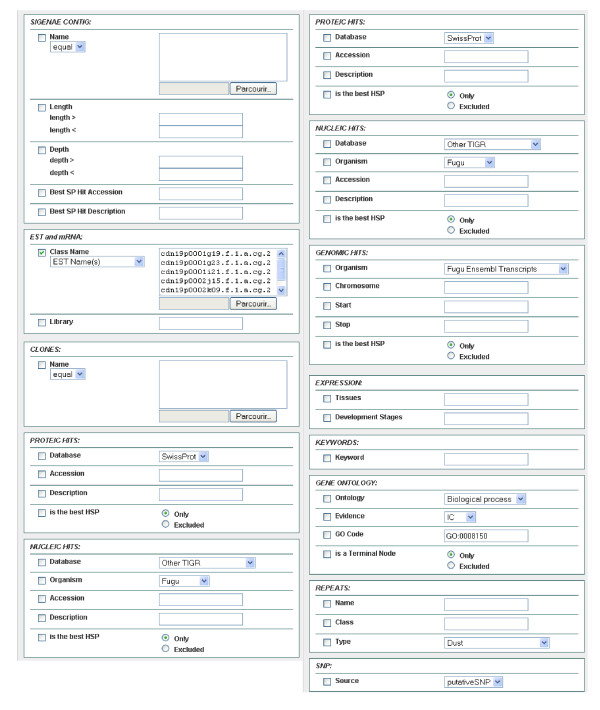
**Filter page available with BioMart in GigasDatabase**. Filter criteria are deposited in blocks. The name is in the upper left corner of the block, and this section contains a list of elements that can be used for selection, corresponding to one table in the database structure. The filter criteria can be based upon contigs, EST and mRNA, clones, protein hits, nucleotide hits, genomic hits, expression, keywords, gene ontology, repeats, and SNP.

**Figure 4:**
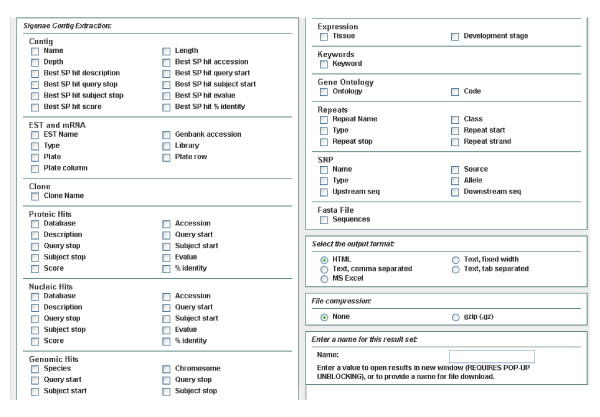
**Output page available with BioMart in GigasDatabase**. Once the filters have been selected, it is possible to select elements for output. For example, best SwissProt (SP) description, best SP hit score, or best SP hit E-value can be exported in several output formats (HTML, Text, MS Excel).

To determine the putative identities of the assembled contig and singleton sequences, we performed BlastX similarity searches on several protein databases. Of the 29,745 unique sequences, 12,790 (43%) had significant matches (E-value < 10^-6^) in the non-redundant protein database. This might be considered as low and due to ESTs within 3' untranslated regions (UTR) that can not be matched to protein sequences, and to the relatively short sequences (about 360 bp) obtained from the SSH libraries. Efforts should be made to generate complete cDNA sequences in *C. gigas *to provide a greater level of assessment of this organism's gene contents and similarities to various other species in the evolutionary spectrum. The complete list of these annotated sequences is reported in Additional File 1. All annotation data have been organized in the user-friendly GigasDatabase using "BioMart", which provides several tools to search cleaned and assembled ESTs. The user may input and submit several filters, such as contig names, EST names, protein hits, nucleotide hits, tissues of expression, as well as keywords, to the server using the web interface, as presented in Figure 3. Once the filters have been selected, it is also possible to select elements for the output files by checking the corresponding boxes in the output data blocks, as shown in Figure 4.

### 4. Gene ontology annotation

More-detailed, functional annotation was performed with BlastX using KAAS (KEGG Automatic Annotation Server: http://www.genome.jp/tools/kaas/). GO categories were successfully assigned to 7,733 (26%) unique ESTs. This low percentage of GO assignment has also been reported previously in *C. gigas *[24] and is probably linked to a high level of amino-acid sequence divergence between marine bivalves and the reference taxa currently used in genomics (such as *Drosophila *(FlyBase) and *Caenorhabditis *(WormBase)), and also to the relatively small average length of the ORFs. Table 4 shows the percentage distribution of gene ontology terms among the 7,733 annotated ESTs. The largest number of annotated sequences was found for a final GO term "Metabolism," which represents 42% of the annotated ESTs among all GO categories. Within this category, the higher GO terms were Amino Acid, Carbohydrate and Lipid Metabolism, with 8, 7.6 and 6.3% of the annotated ESTs, respectively. For "Genetic Information Processing", 4.3 and 4.8% of the annotated ESTs were associated with translation and folding, and sorting degradation respectively. Many ESTs (10.7%) were linked to signal transduction, in the "Environmental Information Processing" category. Finally, Cell Communication (7.0%), Endocrine System (7.9%), and Immune System (4.9%) were the most abundant "Cellular Processes" subterms.

**Table 4:** Gene ontology annotation using the KEGG Automatic Annotation Server for the unique *Crassostrea gigas *sequences from the GigasDatabase.

Categories	Number ESTs	%
**Metabolism**	**3244**	**41.9**
Carbohydrate Metabolism	590	7.6
Energy Metabolism	302	3.9
Lipid Metabolism	484	6.3
Nucleotide Metabolism	153	2.0
Amino Acid Metabolism	619	8.0
Metabolism of Other Amino Acids	171	2.2
Glycan Biosynthesis and Metabolism	273	3.5
Biosynthesis of Polyketides and Nonribosomal Peptides	4	0.1
Metabolism of Cofactors and Vitamins	173	2.2
Biosynthesis of Secondary Metabolites	117	1.5
Xenobiotics Biodegradation and Metabolism	355	4.6

**Genetic Information Processing**	**954**	**12.3**
Transcription	76	1.0
Translation	335	4.3
Folding, Sorting and Degradation	370	4.8
Replication and Repair	173	2.2

**Environmental Information Processing**	**1096**	**14.2**
Membrane Transport	82	1.1
Signal Transduction	830	10.7
Signaling Molecules and Interaction	184	2.4

**Cellular Processes**	**2439**	**31.5**
Cell Motility	188	2.4
Cell Growth and Death	324	4.2
Cell Communication	544	7.0
Endocrine System	611	7.9
Immune System	381	4.9
Nervous System	160	2.1
Sensory System	90	1.2
Development	137	1.8
Behavior	3	0.0

**Total**	**7733**	**100**

"Number ESTs" indicates the number of ESTs associated in the corresponding Gene Ontology, and "%" the corresponding percentage. 100% was established as the total number of unique sequences (7733) having an assigned gene ontology term.

Among these different subcategories of GO, several ESTs potentially connected to physiological functions of the oyster linked with our subset of interest have been discovered (Table 5). For example, for TGFβ signaling regulating a variety of important processes, two new ligands, activin/myostatin and inhibin-like (Table 5), were identified that complete the already-large panel of ligands in *C. gigas *[11,37,38]. Indeed, the identification of new, potential members of the TGFβ superfamily contributes to the TGFβ signaling pathway being recognized as one of the best- characterized systems at the molecular level within lophotrochozoans.

**Table 5:** Selection of some candidate *Crassostrea gigas *ESTs similar to genes potentially involved in some physiological regulatory networks.

	Accession No	Best hit description
**Endo- paracrine controls**	CU997995	activin/myostatin like
	CU984230	inhibin bA like
	CU998397, CU990571	smad
	CU994284, CU991852, CU987529, CU984099, CU991056	follistatin 1
	CU998185, CU991852, CU983909, CU993729, CU987283, CU993729, CU988008	thrombospondin
	FP011148	cysteine rich bmp regulator 2
	CU997999	tolloid-like protein

**Energy metabolis m/reproduct ion**	FP001644, FP008029, FP008849, FP002487, CU984370, CU989738, CU997056	c1q-like adipose specific protein
	FP000698	leptin receptor overlapping transcript-like 1
	FP010154, FP001573, CU993420, CU991531	ovary-specific c1q-like factor
	FP008650	phosphatidylinositol 3-kinase p110 beta
	CU994294, CU990696	acetyl-coenzyme a carboxylase alpha
	CU994253	adiponectin receptor 1
	CU993735	camp-dependent protein kinase
	CU993270	carnitine o-acyltransferase
	CU983945, FP005412, CU994912	neuropeptide y
	CU991233, CU682842	sterol regulatory element binding factor 1

**Immunity**	FP006535, FP010171, CU984422, FP005108, CU998652	caspase
	CU683654	cactus
	CU999108, CU988309, CU993827	myeloid differentiation primary response gene
	FP004666, FP011576, FP002604, CU995719	toll
	CU989449, FP009504, CU998458, CU988135	kappa-b
	CU684230	big defensin
	FP010905	gigasin 2 protein
	FP000856, FP003629, FP006010, FP006279, CU994639	lbp bpi
	FP005503, CU999465, FP006037, FP011761	lipopolysaccharide binding protein
	CU983947, CU996720, FP002226	lps-induced tn factor

Concerning the allocation of energy to reproduction, which may play a crucial role in the ability of oysters to survive summer mortality [13,17], relevant genes potentially involved in the signaling pathway linking reproduction to energy balance have been retrieved, such as ESTs encoding PI3-kinase catalytic subunit beta enzyme (phosphatidylinositol 3-kinase beta, Table 5), leptin receptor, adiponectin-like (ovary-specific c1q-like factor, c1q-like adipose specific protein, Table 5) and a neuropeptide Y ligand (Table 5). Such signaling molecules were recently identified in vertebrates and have important regulatory effects on reproduction [39,40]. For example, leptin and adiponectin were reported to promote fecundity and the growth of germinal cells by increasing the utilization of oxidizable sources. At the opposite, neuropeptide Y inhibits reproduction when energy storage is deficient [41,42].

Finally, concerning the innate immunity of *C. gigas*, new immune-system components have been identified, including signal-transduction elements, LPS binding proteins, antimicrobial peptides, and various protease inhibitors. In particular, a new component of the NF-κB pathway [43], a Toll receptor-like protein, has been sequenced. The most significant feature of the NF-κB pathway is the central role of the NF-κB family in transcriptional activator proteins, ubiquitously expressed and involved in wide variety of biological processes, including inflammation, cell proliferation and differentiation in mammals, as well as development in insects [44].

Further functional studies will be necessary, however, to demonstrate the involvement of these annotated ESTs in the different physiological processes of *C. gigas*. Indeed, this work encourages the use of functional studies, such as RNA interference [45], to ascertain the functions of these genes.

### 5. *in silico *markers

We identified a total of 208 *in silico *microsatellites among the 29,745 unique EST sequences. Most microsatellites were dinucleotide repeats (158) followed by trinucleotides (22) (Table 6 and Additional File 2 for more details). Of the 208 EST-containing microsatellites, only 25 (12%) have significant matches with available ESTs in the NCBI non redundant database whereas 173 (83.2%) have sufficient flanking sequences for primer design. From these, we have recently developed 18 microsatellite markers [22] and have successfully used them for genetic linkage mapping and QTL analysis. Many potentially-useful microsatellites, identified *in silico*, still need to be developed to become useful polymorphic markers for comparative mapping, marker-assisted selection, and evolutionary studies [46]. Single nucleotide polymorphisms (SNPs) have recently become the marker type of choice for linkage and QTL analysis [47]. In most cases, SNPs have relied upon genomic sequencing, BAC end sequencing, or targeted SNP detection. We identified a total of 7,530 putative SNPs, including 1,344 non-synonymous and 5,097 synonymous mutations, and 1,089 indels (Table 7). These SNPs represent an average of 1 SNP per 75 base pairs, slightly lower than the previously-reported frequency of one SNP every 60 base pairs in coding regions [19,48], but higher than in *C. virginica*, with one SNP for every 170 base pairs [49]. These SNPs will also be useful for linkage mapping and population-level studies, and a few to detect selective effects in coding regions of genes regulated by environmental factors. To provide some assessment of the SNPs, the putative SNPs were categorized based upon contig sizes. As mentioned in a previous report [49], the larger the number of sequences involved in a contig, the more likely the SNP can be checked as to whether the putative SNPs represent sequence errors or real SNPs. As shown in Table 8, 2,077 putative SNPs were identified from contigs with only two sequences; 1,358 putative SNPs were identified from contigs with three sequences; 1,044 putative SNPs were identified from contigs with four sequences, and 3,051 putative SNPs were identified from contigs with five or more sequences (Table 8). Consequently, validation and polymorphism analyses must be performed before these putative SNPs can be used because a large proportion of SNPs were identified from contigs with just a few sequences and may be sequencing errors.

**Table 6:** *in silico *microsatellite (Msat) mining of the GigasDatabase.

	Number of Msat	Percentage
Total	208	100
Dinucleotide	158	76.0
Trinucleotide	22	10.6
Tetranucleotide	18	8.7
Pentanucleotide	10	4.8

Percentage represents the percentage of each kind of repeated motif.

**Table 7:** Putative Single Nucleotide Polymorphism (SNP) identification from the GigasDatabase.

SNP Type	Number	Percentage
Non Synonymous	1344	17.85
Synonymous	5097	67.69
Indel	1089	14.46

Total	7530	100

A/G	1860	36.49
T/C	1494	29.31
A/C	551	10.81
A/T	284	5.57
T/G	381	7.47
G/C	518	10.16
TriNucleotides	9	0.19

Total	7530	100

Indels are Insertions and Deletions detected in the sequences with a range of 1 to 13 bases

Two distinct subcategories of SNP have been identified:

- Transition (t_s_) *e.g*. A⇔G; T⇔C that are mutations between two puric bases (A/G) or between two pyrimidic bases (C/T)

- Transversion (t_v_) *e.g*. A⇔C; A⇔T; T⇔G; G⇔C that are mutations between puric and pyrimidic bases

Trinucleotides are polymorphic sites that show three alleles at the same locus.

**Table 8:** Putative SNP distribution in contigs with various number of ESTs.

	Number of contigs	Putative SNP sites
with > 50 sequences	24	432
with 11–50 sequences	433	721
with 6–10 sequences	1352	1663
with 5 sequences	647	235
with 4 sequences	1753	1044
with 3 sequences	1145	1358
with 2 sequences	2586	2077

Total	7940	7530

## Conclusion

In the present paper, we report the production and the sequencing of clones from 9 cDNA libraries derived from different *C. gigas *tissues, and from oysters sampled under different conditions, obtaining 40,845 high-quality ESTs that identify 29,745 unique transcribed sequences. Putative annotation was assigned to 43% of the sequences showing similarity to known genes, mostly from other species, in one or more of the databases used for automatic annotation. The high percentage of *C. gigas *ESTs (57%) with no hits in the protein database implies that there is an enormous potential for discovery of new genes in this species, and possibly new gene networks and metabolic pathways. All data on ESTs, clustering, and annotation can be accessed from the dedicated database, GigasDatabase, available at http://public-contigbrowser.sigenae.org:9090/Crassostrea_gigas/index.html. There is a variety of data-access options, such as database searches on annotation including gene assignments and GO terms, as well as access to self-explanatory, web-based detail annotation archive. This large set of well-characterized clones represents a significant addition to the existing genomic resources for oysters. Indeed, *Crassostrea gigas*, which belongs to the Lophotrochozoans, a large but understudied clade of bilaterian animals, represents a rare non-model species for which the genomic resources available will be very important. Several research teams are now using this important sequence information to examine oyster gene-expression profiles under various experimental and environmental conditions.

## Availability and requirements

Project name: the GigasDatabase.

The Oyster EST contig browser aimed to produce and maintain an automatic annotation of Oyster EST libraries established in three consecutive projects, the Marine Genomics Network of Excellence, the European AquaFirst project and the *Crassostrea gigas *Genoscope project.

The Project home page is: http://public-contigbrowser.sigenae.org:9090/Crassostrea_gigas/index.html

Operating system: LINUX.

Programming language: Perl 5.8.

Other requirements: MySQL 5 or higher, Apache 2.

Licence: Apache like License (free software license with no copyleft).

Any restrictions to use by non-academics: no.

## Authors' contributions

EF, PF, CL, PB and AH assisted in data acquisition and analysis. AH, JS, PL, CF, DG, AT, DM, JM, VB, JdL, YG and EB prepared the biological material and did the construction of the libraries. RR, FG and PW were in charge of the sequencing. FM worked on software design, carried out development, implementation and data processing and CK supervised the web design. CS and SL worked on the detection of *in silico *markers. MM and PP coordinated the involved projects. EF, PF, CL, PB and AH were involved in drafting the manuscript and JM, CC, PP, MM, Sl, GD, PL, JdL, YG and VB revised it for important content. All authors read and approved the final manuscript.

## Supplementary Material

Additional file 1 **List of *C. gigas *annotated sequences**. This table lists 12790 non-redundant sequences identifying known *C. gigas *sequences showing significant similarity (E-value < 10^-6^) with predicted proteins from mollusks and other organisms. This table includes the GenBank Accession numbers of the ESTs and corresponding best SwissProt hit descriptions. Click here for file

Additional file 2 ***in silico *microsatellites in *C. gigas *ESTs**. This table lists the 208 ESTs containing *in silico *microsatellites with, for each sequence, the corresponding motif, the number of repeats, the start and the end position, and the sequence of the *in silico *microsatellite. Click here for file
